# The Protective Role of Vitamin K in Aging and Age-Related Diseases

**DOI:** 10.3390/nu16244341

**Published:** 2024-12-16

**Authors:** Julia Kaźmierczak-Barańska, Bolesław T. Karwowski

**Affiliations:** DNA Damage Laboratory of Food Science Department, Faculty of Pharmacy, Medical University of Lodz, ul. Muszynskiego 1, 90-151 Lodz, Poland; boleslaw.karwowski@umed.lodz.pl

**Keywords:** vitamin K, aging, inflammation, oxidative stress

## Abstract

Aging is an inevitable aspect of life, but age-related diseases are not an inseparable part of the aging process, and their risk can be reduced through a healthy lifestyle. Vitamin K has a broader impact than just blood clotting, and yet it remains overshadowed by other vitamins and underestimated by both doctors and consumers. Vitamin K (VK) is a multifunctional micronutrient with anti-inflammatory and antioxidant properties, whose deficiency may cause age-related diseases such as cardiovascular diseases, neurodegenerative diseases and osteoporosis. There is a growing body of evidence supporting the role of vitamin K as a protective nutrient in aging and inflammation. This review summarizes the current knowledge regarding the molecular aspects of the protective role of vitamin K in aging and age-related diseases and its clinical implications.

## 1. Introduction

Although many theories of aging have been proposed, there is currently no consensus on the matter. Many of the suggested mechanisms appear to interact in various ways. There are two primary theoretical approaches to understanding the causes of aging: deterministic and stochastic concepts [1,2]. Deterministic views propose that aging results from biological mechanisms that have been evolutionarily programmed to lead to death in the post-reproductive period. This perspective suggests a natural tendency to eliminate older, weaker, infirm, and sick individuals. In contrast, stochastic models interpret aging as a gradual, progressive, and irreversible set of structural and functional changes. These changes result in a decline in regenerative, adaptive, and reproductive capacities, alongside disturbances in homeostasis. According to this view, the multi-level changes that occur in an aging organism inevitably lead to death, which is neither adaptive nor programmed by nature. Aging occurs, among other things, as a consequence of the accumulation of molecular and cellular damage that occurs over the life of an organism. These changes lead to various impairments, from the cellular to the physiological level, as well as various functional changes [1,3]. They also increase the burden of oxidative stress and inflammation and weaken the immune system, which in turn increases the risk of many chronic diseases [4]. Aging is a complex process that involves the interaction of multiple pathways and mechanisms. Throughout life, an organism is vulnerable to various external and internal factors which can cause molecular damage and impair functioning. For instance, high levels of free radicals, such as reactive oxygen species (ROS), are produced by damaged mitochondria, as well as industrial and radiation or everyday human activities. These increase oxidative stress, which causes oxidative changes in proteins, lipids, and DNA, which in turn are underlying factors in aging and many diseases [4,5].

In addition to oxidative damage, aging is also characterized by low-level but chronic inflammation, known as inflammaging. Over time, the effectiveness of the body’s immune system decreases, and senescent cells are removed less effectively. Senescent cells exhibit the Senescence-Associated Secretory Phenotype (SASP), which involves the abundant secretion of various pro-inflammatory compounds, including cytokines, chemokines, matrix metalloproteinases (MMPs), and regulatory miRNAs, into the tissue microenvironment [6]. The SASP plays a role in the pathogenesis of chronic and age-related diseases. However, the immune response induced by SASP can also have a protective role in increasing the cytotoxicity of immune cells towards cancer cells [7,8]. The release of pro-inflammatory SASPs and the ineffective removal of senescent cells can result in the further release of SASPs and an increase in the senescent cell population, creating a vicious cycle that causes further aging [9]. Subliminal, chronic inflammation reduces tissue integrity and encourages the emergence of age-related pathologies, including type 2 diabetes, osteoarthritis and sarcopenia [10,11,12]. Inflammation has a detrimental effect on glucose homeostasis and increases insulin resistance. It also exacerbates oxidative stress, increasing the secretion of pro-inflammatory cytokines such as IL1, IL6, C-reactive protein (CRP), transforming growth factor-β (TGFβ), and tumor necrosis factor (TNF), which lead to the constant activation of NF-κB and the dysregulation of cell metabolism [13].

High levels of age-related pro-inflammatory markers are observed in most older individuals, even those who are well and not at clear risk of clinically active diseases; however, they do increase the risk of their occurrence. Elevated levels of TNFα and IL1 can affect the effectiveness of insulin, which can eventually lead to insulin resistance and hyperglycemia [14]. This condition generates more ROS and Advanced Glycation End-Products (AGE), which can damage cells. Furthermore, maintaining insulin sensitivity and ensuring the proper functioning of pancreatic beta cells seem to be critical factors for promoting longevity [15]. Moreover, elevated levels of IL-6 or TNF-α affect nutrition control centers, suppress appetite, change sensory sensations, and inhibit muscle protein synthesis, all of which may promote the development of malnutrition [16].

## 2. Vitamin K—Promoting Healthy Aging

Vitamin K is the general name for a group of compounds that contain a 2-methyl-1,4-naphthoquinone ring with isoprenoid (3C) residues attached to them, including [17] vitamin K1 (phylloquinone, phytomenadione), vitamin K2 (menaquinone, MKn), and vitamin K3 (menadione), a synthetic derivative without a side chain, characterized by high biological activity.

Severe vitamin K deficiencies impair the functioning of the coagulation system; however, such conditions are rare and are typically associated with absorption disorders or the use of VK antagonists in pharmacotherapy. Nevertheless, subclinical deficiencies are more frequently noted, and their imperceptible nature is disturbing. While moderate VK deficiencies do not interfere with the prothrombin system, which is essential for survival, they may influence extrahepatic VKD proteins. These have limited access to VK as it is first directed to the liver and then distributed to other organs and tissues. As such, excess VK intake is needed to meet the VK requirement for extrahepatic proteins. It has been recognized that moderate VK deficiencies are associated with the impaired functioning of osteocalcin, potentially increasing the risk of developing bone fragility after menopause, and Mgp protein, which may result in arterial calcification [18]; in addition, a lack of VK can influence the action of Transforming Growth Factor-beta-induced protein (TGFBI), causing genetic instability and an increased risk of cancer development, due to the formation of mitotic spindle abnormalities [19].

Vitamin K (VK) is a crucial cofactor for the enzyme γ-glutamyl carboxylase (GGCK), present in the endoplasmic reticulum of various cell types [20]. The primary function of VK is to help GGCK post-translationally γ-carboxylate specific glutamic acid residues, which are present in numerous vitamin K-dependent (VKD) proteins. This modification enables VKD proteins to bind calcium and facilitates the proper folding of the Gla domain. Additionally, it allows Gla proteins to bind to cell membranes and perform their intended functions effectively. Moreover, in addition to acting as a GGCK cofactor, VK also exhibits GGCK-independent activity by inhibiting oxidative stress and inflammation.

The passage of time weakens the functions of mitochondrial proteins, which is associated with the increased leakage of ROS from the electron transport chain (ETC) and reduced ATP production. Increased ROS levels result in oxidative stress, which intensifies mitochondrial damage and generates inflammation. Inflammation leads to additional damage and disrupts the ETC chain by TNFα [21], which may also stimulate apoptotic pathways and lower mitochondrial density via NFkB activity [22]. Regenerative abilities are impaired and mitochondrial dysfunction deepens. This creates a vicious circle of OS and inflammation, which feed each other.

Studies in drosophila found MK4 to increase the efficiency of the electron transport chain, which facilitates the production of ATP [23]. Further, mitochondrial damage may induce apoptosis due to the release of cytochrome C or apoptosis-inducing factor (AIF). Increased apoptosis contributes to the pathophysiology of CVD or osteoarthritis by creating foci for calcium crystals, leading to the calcification of blood vessels. Studies on human vascular smooth muscle cells (VSMC) have shown that the induction of apoptosis with anti-Fas antibodies increases calcification 10-fold [24]. Vitamin K2 has been shown to significantly inhibit calcification and apoptosis in rat VSMC cells, via the Gas6 protein [25].

In addition to its protective effect on mitochondria, vitamin K can directly limit oxidative stress by scavenging ROS. The reduced form of vitamin K protects the phospholipids of cell membranes against peroxidation and has 10–100 times greater antioxidant potential than that of α-tocopherol, as noted in vitro [26,27].

Vitamin K may also exert its antioxidant effect indirectly. VK was found to inhibit lipoxygenase 12/15 (Lox12/15) in neuronal cells and oligodendrocytes. Lipoxygenases are ubiquitous enzymes that oxidize unsaturated fatty acids. In oligodendrocytes or neurons, these enzymes are involved in the regulation of neuroplasticity, and any disruption of their function may result in neurological disturbances. Lipoxygenase 12/15 catalyzes the oxidation of arachidonic acid (AA) into hydroperoxyeicosatetraenoic acid (HpETE), which is then reduced to the more stable hydroxyeicosatetraenoic acid (HETE) by glutathione peroxidase. HETE is a lipid mediator of inflammation, the formation of which is associated with the depletion of the cellular GSH pool [28].

Exposure to AA causes death in oligodendrocytes due to severe oxidative stress; however, this effect was inhibited by both VK K1 and MK4. In addition, VK inhibited the activation of Lox12/15 and exerted a protective antioxidant effect by blocking the formation of ROS [29,30]. Additionally, it leads to the protection of the main cellular antioxidant—GSH [27]. Moreover, studies conducted in mice suggest that the blockade of 12/15Lox in the central nervous system may be an effective way of preventing or treating Alzheimer’s disease [31]. Inhibiting Lox can also have anti-inflammatory effects, as lipoxygenases play a key role in modulating tissue inflammation [32]. It has been shown that 12-lipoxygenase (12-Lox), activated by a high-fat diet, is involved in the development of diabetes, causing beta cell dysfunction, oxidative stress, and inflammation [33], through the pro-inflammatory HETE [34]. Kołakowski et al. report that VK2 redirects fatty acid metabolism towards the deposition of a safe fraction of triacylglycerols by increasing the concentration of anti-inflammatory n-3 polyunsaturated fatty acids [35]. Moreover, VK2 has a threefold anti-inflammatory effect: it reduces the fractions of diacylglycerols, which are the main precursors of inflammatory lipids, it lowers the concentration of arachidonic acid (AA), and it lowers the expression of pro-inflammatory cytokines (TNFα and IL6) during inflammation.

Previous research has indicated that vitamin K might play a role in regulating glucose metabolism, insulin resistance, and the lipid profile in the context of a high-fat diet (HFD) [36]. Studies conducted on mice fed with HFD and given VK1 supplements showed an increased expression of SIRT1 and AMPK proteins and a decreased expression of NFkB and pro-inflammatory IL-6. AMP kinase is involved in the regulation of glucose metabolism, while active SIRT1 has a positive impact on fat metabolism. Furthermore, SIRT1, by inhibiting NFkB, reduces inflammation and the expression of pro-inflammatory cytokines [37]. These findings suggest that vitamin K has the potential to lower plasma glucose levels, decrease insulin resistance, and improve the lipid profile. Studies by Oshak et al. also indicate that vitamin K alleviates the inflammatory reaction by inhibiting NFkB and thus lowering the expression of IL6 [38]. Researchers suggest that menaquinone (VK2) reduces the phosphorylation of IKK—a factor that regulates NFkB.

### 2.1. Vitamin K—Reducing the Risk of Age-Related Diseases

**Figure 1:**
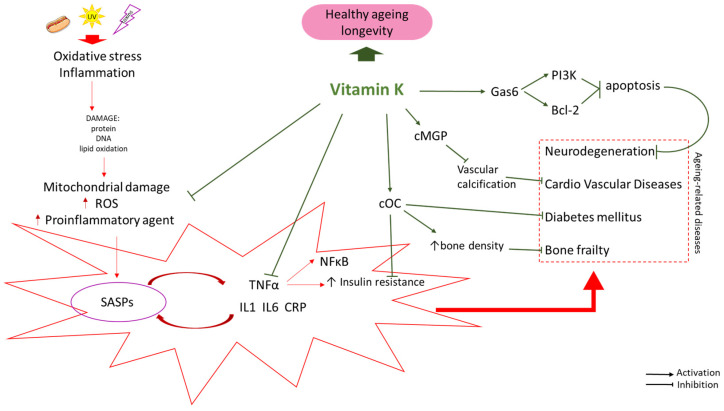
Possible interventions of Vitamin K against aging-related diseases. SASP: Senescence-Associated Secretory Phenotype; UV: ultraviolet; cOC: carboxylated osteocalcin; cMGP: carboxylated Matrix gamma-carboxyglutamic acid (Gla) protein; Gas6: Growth Arrest Specific 6; PI3K: Phosphoinositide 3-kinase; Bcl-2: B-cell CLL/lymphoma 2. Arrows indicate stimulation ↑ or inhibition ↓.

Vitamin K plays a crucial role in maintaining the proper functioning of key proteins that improve bone health, cardiovascular health, blood clotting, and brain function and reduce inflammation. Therefore, it is essential to include vitamin K in any comprehensive plan for healthy aging (Figure 1).

### 2.2. VK Improves the Sensitivity of Cells to Insulin

Significantly lower plasma VK1 levels were noted in patients with type 2 diabetes (T2D) compared to age-matched controls. VK1 levels in T2D were also significantly and inversely associated with fasting glucose and insulin resistance, i.e., the homeostatic model assessment of insulin resistance (HOMA-IR) [36]. A placebo-controlled trial found that vitamin K2 supplementation (30 mg) for four weeks increased insulin sensitivity in healthy young men. The authors attribute the protective effect of vitamin K on glucose metabolism and pancreatic β cell functions to the presence of carboxylated osteocalcin (cOC) [39]. The results of another randomized clinical trial based on T2D patients, characterized by elevated cOC levels, suggest that supplementing with vitamin K2 (100 µg) significantly reduced blood glucose levels and increased insulin sensitivity [40]. Moreover, VK2 reduced the percentage of functional β cells; this is a beneficial effect because increased β cell function is often observed in metabolic syndromes [41].

**Figure 2:**
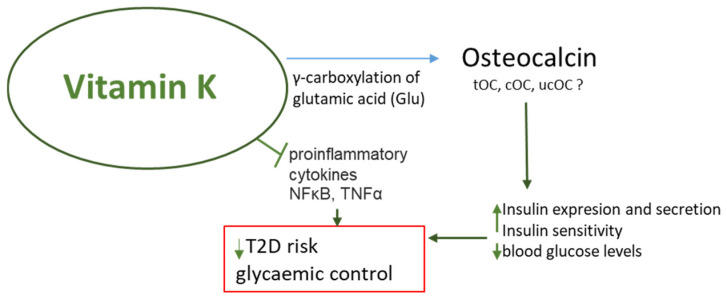
Possible interventions of Vitamin K against type 2 diabetes mellitus. cOC: carboxylated osteocalcin; ucOC: undercarboxylated osteocalcin; tOC: total osteocalcin; T2D: type 2 diabetes. Arrows indicate stimulation/increase ↑ or inhibition/decrease ↓. Red box: pathological/disease conditions.

Osteocalcin is a bone-derived hormone involved in the regulation of energy metabolism. Studies in mice show that osteocalcin knockouts were glucose intolerant and obese, and osteocalcin regulates β cell or insulin expression [42], in which a key role is played by undercarboxylated OC (ucOC) [43]. Numerous rodent studies indicate that ucOC represents a bioactive form of the protein [44]. Lacombe et al. also suggest a role for ucOC in regulating glucose and energy metabolism in humans [45]. Studies conducted in two cohorts using developed, highly specific enzyme-linked immunosorbent assays recognizing human ucOC showed that ucOC is an independent predictor of a fasting glucose concentration. Interestingly, among overweight or obese subjects, those with the highest ucOC levels showed an improved insulin secretion rate and β-cell sensitivity to glucose, suggesting, according to the authors, that ucOC promotes β-cell function. Carboxylation increases the affinity of OC for hydroxyapatite and promotes calcium deposition in bones. UnOC is considered the metabolically active form of OC in mice, but in humans, according to a meta-analysis that included 39 studies with 23,381 participants, both tOC and unOC can be used as biomarkers related to glucose metabolism [46]. Higher serum levels of tOC or unOC were correlated with lower fasting glucose or glycated hemoglobin A1c, suggesting that higher tOC and unOC may be associated with a lower risk of diabetes. Chronic hyperinsulinemia is associated with a higher incidence of fractures [47,48]. One of the most severe complications of T2D is diabetic osteoporosis (DOP). Studies conducted in a T2D mouse model suggest that VK2 inhibited bone loss induced by long-term exposure to high glucose levels and that VK2 treatment was able to restore bone mass; the authors accredit this to the activation of AMPK/SIRT1 signaling by VK2 [49]. However, there is no consistent evidence to clearly support a role for ucOC in the regulation of glucose metabolism in humans (Figure 2).

Vitamin K, as a regulator of osteocalcin function, thus supports pancreas activity, glucose metabolism and, of course, bone strength.

### 2.3. Vitamin K Is a Factor That Reduces the Risk of Osteoporosis

Age is one of the main risk factors for primary osteoporosis [50]. Many studies indicate that the risk of developing osteoporosis is strongly influenced by the level of ucOC. Increased ucOC levels correlate with lower bone mineral density (BMD) values [51,52,53].

**Figure 3:**
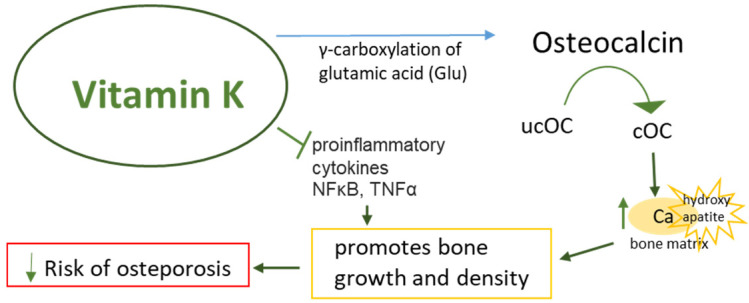
Possible interventions of Vitamin K against osteoporosis. cOC: carboxylated osteocalcin; ucOC: undercarboxylated osteocalcin; Ca: calcium ions. Arrows indicate stimulation/increase ↑ or inhibition/decrease ↓. Red box: pathological/disease conditions.

A meta-analysis of randomized controlled trials (RTCs) examining the effect of VK2 as a dietary supplement on BMD and fracture rates in postmenopausal women (a total of 6425 subjects) [54] found VK2 use to significantly improve lumbar spine BMD (BMD LS) by lowering serum uc-OC levels. Under the influence of VK2 (MK4 or MK7), osteocalcin undergoes γ-carboxylation and is converted into cOC, which may increase bone strength by promoting mineralization. Low serum VK levels and high uc-OC levels are considered risk factors for hip fracture in older women [55,56]. The meta-analysis also suggests that VK2 may reduce the incidence of fractures, but this requires more conclusive data. Another meta-analysis of studies in the Japanese population found that vitamin K2 supplementation plays an important role in maintaining bone mineral density (BMD) and reducing the incidence of fractures in postmenopausal women with osteoporosis. Vitamin K2 high doses (≤45 mg) supplementation appears to be beneficial and safe in the treatment of osteoporosis in postmenopausal women [57]. Interestingly, improving bone metabolism and reducing ucOC can be achieved by supplementing with at least 100 ug MK7 [58]. Providing high concentrations of VK1 and VK2 in the blood is an important factor in reducing the risk of osteoporosis in the elderly and women during menopause, i.e., times when an increase in ucOC concentration is naturally observed. However, vitamin K, especially MK7, may also support bone health through an OC-independent mechanism [59]. This novel mechanism indicates both the pro-anabolic and anti-catabolic activity of VK2 on bone. Yamaguchi et al. demonstrated that MK7 modulates osteoblastogenesis and osteoclastogenesis toward bone formation and the suppression of resorption by antagonizing NFkB activation (Figure 3).

### 2.4. Vitamin K and the Risk of Cardiovascular Diseases

Research suggests that Vitamin K may help protect against the progression of vascular calcification [60], which is linked to a higher risk of cardiovascular disease (CVD). This protective effect is attributed to the presence of matrix gamma-carboxyglutamic acid (Gla) protein (MGP) vitamin K-dependent proteins in the vascular tissue. Nonphosphorylated-undercarboxylated MGP (dp-ucMGP) is strongly associated with the vascular calcification of various etiologies; this suggests that vitamin K may play a role in inhibiting calcification of the intimal and medial membrane of blood vessels [61]. The PREDIMED (Prevención con Dieta Mediterránea) study, a prospective cohort analysis of 7216 participants focusing on a Mediterranean population at a high risk of cardiovascular disease, found that a higher intake of VK1 reduced the risk of CVD-related death [62]. Similarly, the Perth Longitudinal Study of Aging Women (PLSAW), after 15 years of follow-up of 1436 older Australian women, found that women with the highest VK intake (119.3 μg/day) had a lower relative risk of all-cause mortality and CVD mortality than those with the lowest vitamin K1 intake (49.1 μg/day) [63]. However, studies on the association between vitamin K intake and CVD risk [64,65,66] have limitations and may not be entirely reliable, as data estimated using the food frequency questionnaire do not accurately represent the true state of VK in the participants [67]. Concluding causality from observational studies is difficult. Randomized controlled trials will provide more robust information. A systematic review analyzed the results of nine RCTs to investigate the impact of vitamin K supplementation on surrogate measures of cardiovascular disease (CVD), such as vascular calcification, vascular stiffness, and intimal thickness [68]. A total of 1589 patients, aged 55 to 80 years, were included and given K1 (500 µg to 2 mg daily) or MK7 (90 μg to 2000 μg daily) from 6 months to 3 years. The quality of the studies was rated low. According to the authors, the results available from the RCTs do not provide evidence that vitamin K supplementation prevents worsening surrogate measures of CVD. Dp-ucMGP levels were also analyzed because high levels may predict and increase the risk of cardiovascular events. The results indicate that while MK7 supplementation effectively reduced dp-ucMGP levels, the improvement in dp-ucMGP does not translate into reduced vascular calcification. The authors conclude that despite the potential benefits of vitamin K treatment, there are no well-conducted and adequately powered studies. However, a recent post hoc analysis of a double-blinded, randomized, placebo-controlled trial (n = 149) using 18F-NaF PET imaging in diabetic patients showed that supplementation with 10 mg/d of vitamin K1 may prevent the development of new calcified lesions in the aorta and coronary arteries [69]. Nevertheless, results from large RCTs with hard CVD endpoints are still needed.

### 2.5. Vitamin K Supports Brain Health and Reduces the Risk of Cognitive Impairment

Cognitive impairment (CI) is a chronic condition characterized by a loss of memory, concentration, and impaired ability to learn new things. It affects the quality of everyday life. Up to one-third of cases of CI result in dementia and may result in the inability to live independently. A recent systematic review and meta-analysis estimated that the incidence of CI increases with age [70]. The risk factors for the development of dementia include insulin resistance, inflammation, and oxidative stress, as well as the coexistence of metabolic disorders such as hypertension, hypercholesterolemia, obesity, and diabetes [71,72,73,74,75]. In addition, a meta-analysis estimated that disease states such as type 2 diabetes and obesity may also significantly and independently increase the risk of Alzheimer’s disease [76].

**Figure 4:**
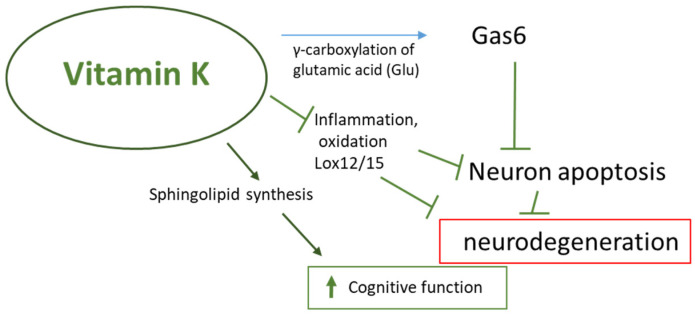
Possible vitamin K interventions supporting brain function. Gas6: growth-arrest-specific gene 6 (Gas6) VK-dependent Gla protein; Lox12/15: lipoxygenase 12/15. Arrows indicate stimulation/increase ↑ or inhibition/decrease ↓. Red box: pathological/disease conditions.

Evidence supports a strong association between healthy dietary patterns, such as the Mediterranean diet, and a lower chance of cognitive impairment [77]. Research suggests that vitamin K may support brain function by participating in the metabolism of sphingolipids, which are the main components of cell membranes. Changes in sphingolipid metabolism have been associated with the onset of neurodegenerative aging processes and neurodegenerative diseases such as Alzheimer’s and Parkinson’s disease [78]. Furthermore, it was observed that Gas-6 (VKDP) could prevent β-amyloid-induced brain cell apoptosis. Gas-6 is widely expressed in the CNS, and to achieve full biological activity, Gas-6 requires vitamin K [79]. Gas6 has been shown to prevent cell apoptosis through the activation of the PI3K-associated protein kinase B (Akt) and Bcl-2-associated death promoter protein (Bad) (PI3K/Akt/Bad)-signaling pathway [80,81]. The pro-inflammatory state and oxidative stress are inherent in the aging process and are strongly associated with cognitive decline and the subsequent development of dementia in older adults. The antioxidant properties of vitamin K have been observed to benefit neuronal cells, especially cultured neurons and oligodendrocytes, preventing cell death caused by oxidative stress (Figure 4) [28,82].

Reduced levels of vitamin K have been reported in patients with Alzheimer’s disease compared to controls [83]. The authors suggest that this may be related to the presence of the APOE4 genotype, which has been associated with a higher incidence of Alzheimer’s disease with age. This could be due to an increased clearance of intestinal lipoproteins rich in vitamin K [84]. Other prospective studies have observed that the use of vitamin K antagonists (VKAs) was associated with lower (i.e., worse) executive function scores in elderly outpatients; VKA use among geriatric outpatients has also been found to result in the frequent and more severe deterioration of executive functions [85]. Importantly, studies in mice indicate that VKA treatment resulted in a decrease in MK4 (the most abundant form of VK in the brain) together with a high phylloquinone concentration, with the concomitant deterioration of cognitive and behavioral functions [86]. Post-mortem studies in 325 deceased participants of the Rush Memory and Aging Project found that higher brain concentrations of MK4 were associated with a lower likelihood of developing dementia or mild cognitive impairment, along with lower global Alzheimer’s disease pathology scores; in addition, higher brain concentrations of MK4 were associated with a 17% to 20% lower likelihood of developing dementia or mild cognitive impairment and with lower global Alzheimer’s disease pathology scores [87]. It has been proposed that chronically low vitamin K levels may be a risk factor for Alzheimer’s disease and that vitamin K supplementation may have a beneficial effect in preventing or treating this disease [88]; however, this theory still needs to be investigated in large, well-designed studies aimed at confirming the neuroprotective potential of vitamin K.

## 3. Vitamin K Status and Biomarkers

According to the European Food Safety Authority (EFSA), Vitamin K is present in two forms in food: K1 phylloquinone and K2 menaquinones (MKn). However, as there are no effective markers to determine vitamin K intake and status in the body, no dietary reference values (DRVs) have been established [89]. Instead, adequate intake (AI) values of 70 ug daily apply only to phylloquinone, a form of VK that is primarily found in the liver and heart but is present in all tissues. There are no dietary recommendations for VK2. The human gut microbiota produces VK2 (MKn), but bioavailability is limited because the molecules remain largely associated with the microbial cell membrane [20,90]. Although mammals have developed an efficient system for recycling vitamin K, compared to other vitamins, the intake of vitamin K is very low, considering how little vegetables are included in the daily Western diet. The liver, the site of the synthesis of coagulation factors, is able to extract vitamin K from the circulation very efficiently [91]. Therefore, it is questionable whether the current daily intake of vitamin K is sufficient to meet the requirements of extrahepatic tissues. It is important to note that different forms of vitamin K are significant and tissue-specific. K1 is primarily associated with the liver, while MK4 predominates in the vascular walls, brain, and pancreas. [92,93]. According to a recent landmark stable isotope study in rodents, phylloquinone is also detected in extraintestinal and extrahepatic tissues, whereas MK4 is present in all tissues and is the only form present in the brain and pancreas [94]. This is due to a different distribution profile: K1 is mainly associated with the triacylglycerol-rich lipoprotein fraction (TGRLP), while menaquinones are present in the LDL fraction and MK4 in HDL [20]. The well-established physiological function of vitamin K is as a cofactor for the enzyme gamma-glutamylcarboxylase (GGCX), with all forms of vitamin K being able to act as a cofactor for GGCX [95]. As suggested by the results of an RCT obtained in 365 postmenopausal patients, both K1 and MK4 have similar efficacy as cofactors for GGCX. In a study that tested the efficacy of osteocalcin carboxylation, both phylloquinone and MK4 treatment rapidly reduced % circulating ucOC [96].

MK7, another form of VK2 found in fermented natto soy, has the highest bioavailability [27,97,98] and is maybe about 2.5 times more potent than phylloquinone in stimulating c-carboxylation [99,100]. Studies using isotopic forms of vitamin K have shown that both VK1 in the diet and various forms of MKn serve as precursors of MK4 in the tissue [94]. It is thought that K1 may be converted by UbiA prenyltransferase containing 1 (UBIAD1) to produce MK4. UBIAD1 is an enzyme that is found throughout the body and has MK4 biosynthetic activity in most tissues [101]. The predominant form consumed and present in plasma in the general population is K1 [102]; however, MK7 predominates in the Japanese population [103,104,105]. As the more senior section of Japanese society is renowned for their good health, it could be the case that MK7 may play an important role in healthy aging and longevity.

Although severe vitamin K deficiency is rare, people on a restricted diet or malnourished, people suffering from lipid malabsorption, cancer, or kidney disease, newborns, and the elderly are at an increased risk of vitamin K deficiency. Various indicators are used to assess vitamin K levels; the direct method of assessing vitamin K status is the analysis of vitamin K1 concentration in plasma, where concentrations < 0.15 µg/L indicate deficiency [106]. According to EFSA, the content of phylloquinone in the body of about 0.55 mcg/kg of body weight in healthy adults at a steady state is not associated with symptoms of vitamin K deficiency. As explained by the EFSA Panel, assuming that the absorption of phylloquinone from the European diet will be about 35%, phylloquinone intake at the level of 1 µg/kg of body weight per day in the European population is optimal for satisfying the γ-carboxylation of hepatic vitamin K-dependent proteins (VKDP) and balancing losses resulting from micronutrient turnover in the body [17]. The measurements of circulating vitamin K recorded in studies are often not validated using external quality assurance programs. As such, there is a need for a unified and standardized system for assessing vitamin K in blood to improve the comparability of tests. Card et al. report that low phylloquinone concentrations frequently yield false-positive results [107]. As such, many studies could have incorrectly estimated the VK status, and a more accurate analysis may indicate a suboptimal level of vitamin K. Currently, reference materials are available in the K1 standard, developed in 2009 (KEQAS SRM-001) and 2019 (KEQAS SRM-002), and KEQAS participation should be reported in all scientific reports [108].

While it is important to assess circulating vitamin K levels, studies should include an analysis of markers of vitamin K functionality, such as the PIVKA-II (protein induced by the lack of vitamin K or antagonists), as these can better reveal both vitamin K levels and utilization [109]. PIVKA-II is detectable at very low concentrations in the plasma of healthy individuals and increases when there is insufficient vitamin K in the diet (reference range 17.4–50.9 mAU/mL) [100]. PIVKA-II might serve as a valuable biomarker of suboptimal vitamin K status, especially in the chronic kidney disease population, because it responds in a dose- and time-dependent manner to vitamin K supplementation, and its measurement is independent of renal function [110,111,112]. However, PIVKA-II is a marker reflecting liver VK stores, and it is also necessary to consider the functionality of extrahepatic proteins; in this case, interesting options include osteocalcin [113] and MGP, which occur in non-carboxylated or undercarboxylated forms in conditions of VK deficit [114] and are indirect indicators of the assessment of vitamin K status. High plasma dp-ucMGP concentrations reflect low vitamin K levels and are associated with vascular calcification. A study of 491 patients using commercial immunoassays confirmed that dp-ucMGP is an effective functional biomarker of vitamin K status [115], sensitive to vitamin K supplementation [68,116,117]. The cOC and ucOC ratio is a widely used marker of bone metabolism, as well as a biomarker of glucose metabolism [46], and the ucOC level is a clinical marker of VK deficiency in osteoporosis therapy and is a good predictor of fracture risk [51].

## 4. Summary

Aging is an inevitable aspect of life, and it is a natural process that occurs in a healthy body without any major complications. However, it is crucial to ensure its proper and uninterrupted course. Age-related diseases are not an inseparable part of the aging process, and their risk of occurrence can be reduced through a correct and conscious lifestyle. This reduction is essential because these diseases decrease the quality of life and are not only a problem for the patient but also for their family and community. To ensure healthy aging, promoting activity and independence, maintaining a balanced diet and regular physical activity is crucial. A specific nutrition model for longevity has not yet been recognized; however, longevity is modulated at the molecular level by micronutrients.

Unfortunately, this stage of life is characterized by both social and physiological changes which can lead to malnutrition. A well-balanced diet providing sufficient nutrients promotes healthy aging and reduces the risk of disease. Geriatric syndromes, such as frailty, are partly caused by a shortage of polyunsaturated fatty acids (PUFAs), vitamins, and micro- and macroelements. As such, consistently meeting the body’s micronutrient needs is a key element of healthy aging. When their supply is insufficient, their use is primarily determined by a triage mechanism [118]; in such cases, the body reserves micronutrients for critical processes that enable immediate survival, drawing them away from processes such as repairing damage that determine longevity in the long run. Unfortunately, the typical high-calorie Western diet is unbalanced and low in micronutrients; leading to moderate deficiencies with no clinical symptoms. This lack of symptoms is often ignored and downplayed by consumers.

Nutrition research based on the assumption that consuming products believed to contain VK is sufficient to fulfill all functions is not highly reliable. The lack of specific information about the exact levels of VK in different products and the use of subjective assessments of VK consumption make it difficult to compare results from various studies. There is a need for well-designed and repeatable studies that determine accurate VK levels using biomarkers and consider the true impact of different forms of vitamin K supplementation. Nevertheless, emerging information suggests that vitamin K has great potential for promoting health and healthy aging, despite often being overlooked in favor of other vitamins. The role of vitamin K (VK) in aging and age-related diseases is primarily due to its anti-oxidant and anti-inflammatory effects. VK plays a crucial part in bone metabolism by carboxylating osteocalcin, a protein that supports calcium transportation and storage in bones. VK helps prevent calcium buildup in blood vessel walls by activating matrix Gla protein, potentially reducing the risk of cardiovascular disease and possibly counteracting pathogenic renal calcification. There is emerging evidence that VK supplementation in patients with chronic kidney disease may be associated with some health benefits [119]. Additionally, VK may lower the risk of metabolic disorders such as type 2 diabetes by enhancing insulin sensitivity and exerting anti-inflammatory effects [122, 123}. VK is also involved in brain health and may help mitigate cognitive decline by carboxylating the Gas6 protein, VKDP, which could protect against neuronal apoptosis induced by oxidative stress and amyloid beta. A summary of the role of VK in aging is presented in Table 1.

This demonstrates the significant health and anti-aging potential of vitamin K. However, the requirements for VK intake vary based on diet, vitamin K form, age, and the presence of chronic or metabolic diseases. It is insufficient to rely solely on a diet rich in green leafy vegetables for adequate VK intake. There is a need to establish precise recommended doses not only for K1 but also for VK2 (MK4 and MK7), which possess varying levels of bioavailability. This is especially crucial for older individuals at a higher risk of metabolic diseases.

## Figures and Tables

**Table 1:** The role of VK in aging.

	The Scope of Vitamins K’s Actions	Pro-Health Effect
Brain	↓ ROS ↓ Lox12/15	Neuroprotection Lower risk of neurodegeneration (AD, PD) Improved cognitive function [77,79,82,87]
	↑ Gas6 ↓ neurons apoptosis	
	↓ β amyloid ↑ synthesis of sphingolipids	
Blood vessels	↑ cMGP ↑ cOC ↓ calcification of vessels	Lower risk of calciphylaxis and new vascular and renal calcification * [68,69,119]
Bone structure	↑ cOC ↑ Ca sequestration in the bone matrix ↑ promoting bone mineralisation	Lower risk of osteoporosis Reduced incidence of fractures [54,57]
	↓ bone resorption	
Metabolism of glucose	↓ inflammation ↓ blood glucose level ↓ level of lipids in blood ↑ insulin sensitivity ↑ cOC	Improved insulin sensitivity Reduced insulin resistance Reduced risk of T2D [40,120,121]

* Require more conclusive data. Arrows indicate stimulation/increase ↑ or inhibition/decrease ↓.
